# Resveratrol and Extra Virgin Olive Oil: Protective Agents Against Age-Related Disease

**DOI:** 10.3390/nu16244258

**Published:** 2024-12-10

**Authors:** Evgeny Morkovin, Roman Litvinov, Alexey Koushner, Denis Babkov

**Affiliations:** 1 Scientific Center for Innovative Drugs, Volgograd State Medical University, Novorossiyskaya 39, 400087 Volgograd, Russia; litvinov_r@innovvita.com (R.L.);; 2 LLC «InnoVVita», Office 401, Room 2, 6 Komsomolskaya St., 400066 Volgograd, Russia; 3 Research Laboratory of Medical Imaging, Institute for Advanced Training of Medical Personnel, St. F. Engelsa, 58A, 394036 Voronezh, Russia

**Keywords:** resveratrol, extra virgin olive oil, age-related diseases, Mediterranean diet

## Abstract

Resveratrol and extra virgin olive oil are both recognized for their potential protective effects against age-related diseases. This overview highlights their mechanisms of action, health benefits, and the scientific evidence supporting their roles in promoting longevity and cognitive health. A literature search was conducted. Important findings related to the health benefits, mechanisms of action, and clinical implications of resveratrol and EVOO were summarized. Both resveratrol and EVOO have complementary mechanisms that may enhance their anti-aging effects. Resveratrol and EVOO are promising age-related disease-protective agents. Their antioxidant, anti-inflammatory, and neuroprotective properties contribute to improved health outcomes and longevity. Incorporating these compounds into a balanced diet may offer significant benefits for aging populations, supporting cognitive health and reducing the risk of chronic diseases. Continued research is essential to fully understand their mechanisms and optimize their use in clinical settings. Future research should focus on investigating the synergistic effects of resveratrol and EVOO when consumed together, as they may enhance each other’s bioavailability and efficacy in promoting health; conducting extensive clinical trials to confirm the long-term benefits of these compounds in various populations, particularly in aging individuals; further exploring the molecular pathways through which resveratrol and EVOO exert their effects, including their interactions with gut microbiota and metabolic pathways.

## 1. Introduction

Resveratrol is a polyphenolic compound found in various foods, particularly in grapes, red wine, and berries. It exhibits multiple bioactivities, including antioxidant, anti-inflammatory, and neuroprotective effects, making it a promising agent for combating age-related diseases [1,2]. Resveratrol scavenges free radicals and enhances the activity of endogenous antioxidant enzymes. This action protects cellular components from damage associated damage and helps reduce oxidative stress, a significant factor in aging and the development of age-related diseases, such as cardiovascular diseases and diabetes [1].

Resveratrol garnered attention for its ability to activate sirtuins, a group of proteins linked to longevity and metabolic regulation. Resveratrol activates NAD^+^-dependent deacetylase sirtuin-1 (SIRT1), a protein involved in regulating cellular processes such as metabolism, inflammation, and stress resistance. This activation mimics the effects of caloric restriction, a well-documented method for extending lifespan in various organisms. Resveratrol’s antioxidant properties help reduce oxidative stress, a significant factor in aging and the development of age-related diseases, such as cardiovascular diseases and diabetes [2,3]. By inhibiting pro-inflammatory pathways, resveratrol can reduce chronic inflammation, a significant contributor to age-related diseases like cardiovascular disease and neurodegeneration [1,2].

Studies have shown that resveratrol can improve cognitive function and reduce the risk of neurodegenerative diseases. For example, clinical trials demonstrated that resveratrol supplementation may enhance memory performance and functional connectivity in the brains of older adults [4]. Research indicates that resveratrol can improve metabolic health by lowering blood glucose levels and enhancing insulin sensitivity. In clinical trials, supplementation of approximately 1 gram per day has shown the potential to reduce glucose levels significantly. However, while animal studies suggest benefits in lifespan extension, human studies are still limited, and further research is needed to confirm these effects in humans [5].

Resveratrol faces limitations in its activity and pharmacokinetics due to factors like low aqueous solubility, oral bioavailability, and chemical instability [6]. These challenges hinder its effectiveness in nutritional and pharmaceutical applications. To address these limitations, recent strategies have focused on developing delivery systems based on olive oil, which can enhance the bioavailability and efficacy of resveratrol. Olive oil, particularly EVOO, contains phenolic compounds that contribute to health benefits. Nano and microencapsulation methods using olive oil have been explored to stabilize resveratrol and improve its delivery, overcoming issues like low solubility and stability [7,8,9]. By utilizing olive oil as a carrier, these delivery systems aim to enhance the absorption and bioavailability of resveratrol, potentially maximizing its therapeutic effects [10,11].

EVOO is a cornerstone of the Mediterranean diet, which is associated with numerous health benefits, including increased longevity and reduced risk of chronic diseases [12,13,14,15]. The health-promoting effects of EVOO are largely attributed to its high content of monounsaturated fatty acids (MUFA) and bioactive compounds, particularly polyphenols such as hydroxytyrosol and oleuropein. These components contribute to its health-promoting properties [16,17]. EVOO polyphenols exhibit strong antioxidant and anti-inflammatory activities, which can modulate cellular pathways related to oxidative stress and inflammation—two critical factors in the aging process. Studies have shown that these compounds can activate the nuclear factor erythroid 2-related factor 2 (Nrf-2) signaling pathway, enhancing the body’s defense mechanisms against oxidative damage, while also inhibiting the pro-inflammatory nuclear factor κB (NF-κB) pathway [16,17,18].

Regular consumption of EVOO has been linked to improved cardiovascular health, reduced incidence of neurodegenerative diseases, and overall healthier aging [16]. EVOO consumption has been linked to improved cognitive function and a reduced risk of neurodegenerative diseases. The phenolic compounds in EVOO can help clear amyloid-beta, a protein associated with Alzheimer’s disease, thus potentially slowing cognitive decline [17,19]. Epidemiological studies have shown that adherence to a Mediterranean diet, which is rich in EVOO, is associated with lower mortality rates and reduced incidences of age-related diseases, including cognitive decline [17,19]. Additionally, clinical trials have demonstrated cognitive improvements in older adults consuming EVOO as part of their diet [16].

Resveratrol and EVOO are both recognized for their anti-aging effects, primarily through their antioxidant and anti-inflammatory properties, and both compounds demonstrate significant potential as protective agents against age-related diseases. Their mechanisms primarily involve reducing oxidative stress and inflammation, which are central to the aging process. Incorporating these compounds into a balanced diet may promote healthier aging and reduce the risk of chronic diseases associated with aging [20]. Further research is essential to fully elucidate their effects, particularly in human populations.

## 2. Effects and Mechanisms of Resveratrol

### 2.1. Molecular Mechanism of Resveratrol Action

Resveratrol, a polyphenol found in the skin of red grapes and various other fruits, has gained significant attention for its potential health benefits. While the complete mechanism of resveratrol is still being elucidated, a growing body of evidence suggests that its action is largely mediated through the activation of the sirtuin (SIRT) family of proteins, particularly SIRT1. Resveratrol directly binds to and activates SIRT1, a NAD+-dependent protein deacetylase that regulates various cellular processes related to aging and metabolism [21,22]. The binding of resveratrol to SIRT1 promotes deacetylation of SIRT1 substrates, such as p53, NF-κB, and Pparg coactivator 1 alpha (PGC-1α), leading to modulation of their activities [23,24].

Resveratrol-induced SIRT1 activation mimics the effects of caloric restriction, which has been shown to extend lifespan in various organisms [21,25]. SIRT1 activation by resveratrol leads to the regulation of various cellular pathways, including the inhibition of inflammatory response by deacetylating and inactivating NF-κB [18,23]; improvement of mitochondrial function and energy metabolism by deacetylating and activating PGC-1α [24,26]; induction of antioxidant response through the activation of the Nrf-2 pathway [18]; modulation of cell survival and apoptosis by deacetylating p53 [25]. In addition to SIRT1, resveratrol has been shown to inhibit human SIRT3 and stimulate SIRT5 in a substrate-specific manner [21]. The specific activation or inhibition of different sirtuin isoforms by resveratrol depends on the substrate and the binding interactions between resveratrol, the sirtuin, and the substrate [21,22]. In summary, resveratrol’s mechanism of action primarily involves the activation of SIRT1, which leads to the modulation of various cellular pathways related to aging, metabolism, inflammation, and oxidative stress. However, resveratrol’s effects are substrate-specific and can vary depending on the cellular context and the specific sirtuin isoforms involved.

Resveratrol inhibits the activation of the NLR family pyrin domain containing 3 (NLRP3) inflammasome, which is critical in the development of chronic inflammatory diseases. It suppresses the activation step of the NLRP3 inflammasome by preserving mitochondrial integrity and preventing mitochondrial damage, which is often a trigger for NLRP3 activation. Additionally, resveratrol induces autophagy, a cellular process that helps remove damaged organelles and proteins. This autophagic activity is mediated through the activation of p38 mitogen-activated kinase (MAPK), and macrophages treated with autophagy inhibitors show resistance to the suppressive effects of resveratrol on NLRP3 activation [27]. By inhibiting NLRP3 activation, resveratrol reduces the secretion of interleukin 1β (IL-1β) and other pro-inflammatory cytokines, thereby mitigating inflammation. This action is particularly important in conditions characterized by chronic inflammation, such as metabolic syndrome, cardiovascular diseases, and neurodegenerative disorders [28,29].

Aging is associated with a state of chronic low-grade inflammation, often referred to as “inflammaging”. This condition contributes to the development of various age-related diseases, including Alzheimer’s disease, cardiovascular diseases, and diabetes. Resveratrol’s ability to inhibit NLRP3 inflammasome activation and reduce inflammatory cytokine production can help alleviate the effects of inflammaging [1]. Resveratrol has shown promise in protecting against neurodegenerative diseases by reducing neuroinflammation. In models of Alzheimer’s disease, resveratrol administration has been linked to decreased levels of amyloid-beta and tau proteins, both of which are associated with neurodegeneration. By inhibiting NLRP3 activation, resveratrol may help preserve cognitive function and reduce the risk of developing neurodegenerative conditions [2,29,30].

The anti-inflammatory effects of resveratrol also extend to cardiovascular health. By inhibiting NLRP3 and reducing systemic inflammation, resveratrol can help lower the risk of atherosclerosis and other cardiovascular diseases, which are prevalent in aging populations [1,2,28,31]. Resveratrol has been shown to improve insulin sensitivity and reduce the risk of type 2 diabetes by modulating inflammatory pathways. Its ability to inhibit the NLRP3 inflammasome may play a role in this metabolic protection, making it a potential therapeutic agent for age-related metabolic disorders [2,29]. Resveratrol acts as a potent NLRP3 inflammasome inhibitor, offering significant implications for the management and prevention of age-related diseases characterized by chronic inflammation. By preserving mitochondrial integrity and promoting autophagy, resveratrol not only mitigates inflammation but also supports overall health in aging populations.

Resveratrol acts as a pathway-selective ligand for estrogen receptor-alpha (ERα). This interaction has been shown to modulate inflammatory responses without promoting cell proliferation, distinguishing it from traditional estrogenic activity. The binding of resveratrol to ERα induces a conformational change that alters the receptor’s coregulator recruitment profile, enhancing its ability to suppress pro-inflammatory cytokines like interleukin-6 (IL-6) while avoiding the oncogenic effects typically associated with ER activation [32]. This selective modulation suggests that resveratrol can influence gene expression in a manner that is beneficial for reducing inflammation without the adverse effects linked to estrogen signaling.

Furthermore, resveratrol’s interaction with ERα is intricately linked to its effects on sirtuins. ERα is a substrate for SIRT1, and SIRT1 itself can act as a coregulator for ER signaling. This interplay complicates the understanding of how these pathways interact but also emphasizes the potential for synergistic effects where resveratrol enhances both ER and SIRT1 activity to modulate inflammatory responses and cellular metabolism [32,33].

In addition to its role in modulating ER signaling, resveratrol is known to activate 5′ adenosine monophosphate-activated protein kinase (AMPK), a critical regulator of cellular energy homeostasis. Activation of AMPK by resveratrol has been associated with various metabolic benefits, including improved glucose uptake and lipid metabolism. Resveratrol enhances AMPK activity through mechanisms that may involve increased intracellular calcium levels and subsequent activation of calcium/calmodulin-dependent protein kinase kinase [34]. This pathway is particularly relevant in contexts, such as diabetes and obesity, where AMPK plays a crucial role in regulating metabolic processes.

Moreover, resveratrol’s ability to stimulate AMPK can also lead to downstream effects on mitochondrial biogenesis and oxidative stress response, further contributing to its protective roles against age-related diseases and metabolic syndromes [33,35]. The activation of AMPK not only aids in energy regulation but also intersects with other signaling pathways involved in inflammation and cell survival. The diverse interactions of resveratrol with molecular targets, such as AMPK and estrogen receptors, underscore its potential as a therapeutic agent. By selectively modulating these pathways, resveratrol may offer benefits in managing inflammation, metabolic disorders, and possibly even cancer prevention. As research continues to unravel these complex mechanisms, resveratrol’s role in health promotion and disease prevention appears increasingly promising.

Future research should continue to explore the therapeutic potential of resveratrol, particularly in the context of chronic inflammatory diseases, and investigate effective delivery systems to enhance its bioavailability, while several effective strategies to overcome its inherent limitations like low aqueous solubility, oral bioavailability, and chemical instability were proposed [10,11,36,37,38].

A nanotechnological approach involves encapsulating resveratrol in nanoparticles, which can improve its solubility and stability [6]. By using materials like lipids or polymers, nanoencapsulation can protect resveratrol from degradation and facilitate its absorption in the gastrointestinal tract. Utilizing lipid carriers, such as solid lipid nanoparticles or nanostructured lipid carriers, can significantly enhance the absorption of resveratrol. These systems improve solubility and provide a controlled release of the compound [39,40,41,42].

Combining resveratrol with fatty acids or other lipophilic compounds can enhance its solubility in biological membranes, and this strategy takes advantage of the natural lipid absorption pathways in the body, potentially leading to improved bioavailability [6,41,43,44,45,46]. EVOO has been shown to enhance the bioavailability of resveratrol due to its high content of monounsaturated fatty acids and polyphenols. Furthermore, the content of phenolic compounds in EVOO, in combination with resveratrol, not only improves absorption but may also provide synergistic health benefits. Furthermore, encapsulation methods using olive oil have been explored to stabilize resveratrol and improve its delivery, overcoming issues like low solubility and stability [7,8,9]. Pairing resveratrol with other bioactive compounds that have complementary mechanisms can enhance its overall efficacy and bioavailability. For example, combining resveratrol with curcumin or quercetin may improve absorption and therapeutic effects through various pathways [6,39,47,48,49]. Certain substances, such as piperine (found in black pepper), can also enhance the bioavailability of resveratrol by inhibiting metabolic enzymes that would otherwise degrade it before absorption [43,50,51,52]. Another strategy is to reduce the particle size of resveratrol, which can increase its surface area, enhancing dissolution rates and absorption in the gastrointestinal tract. It is possible to use a spray-drying method to transform resveratrol (either as a single substance or as a part of a combination with an enhancer) into a powder form that is more easily absorbed when ingested, improving its bioavailability compared to traditional formulations [43,53,54,55].

By implementing these strategies, researchers and formulators can significantly improve the bioavailability of resveratrol, maximizing its potential health benefits and therapeutic applications.

### 2.2. Resveratrol in Cognitive Enhancement

Resveratrol has been studied for its potential cognitive enhancement effects, particularly in the context of aging and neurodegenerative diseases. Resveratrol exhibits neuroprotective effects through several mechanisms including antioxidant and anti-inflammatory activity.

It scavenges free radicals and reduces oxidative stress, which is critical in preventing neuronal damage associated with cognitive decline [56]. Resveratrol inhibits pro-inflammatory pathways, reducing the expression of inflammatory cytokines and the activation of NF-κB, which can contribute to neurodegeneration [27,57].

Animal studies have demonstrated that resveratrol can improve learning and memory by modulating neuroprotective pathways and reducing oxidative stress [56]. Several clinical studies suggest that resveratrol may enhance cognitive performance, particularly in specific populations. A 14-week randomized controlled trial showed that low-dose resveratrol supplementation (75 mg twice daily) significantly improved cognitive performance and cerebrovascular function in postmenopausal women [57,58]. Improvements were noted in verbal memory and overall cognitive function, particularly in women aged 65 and older. Another study involving a 26-week supplementation with 200 mg/day of resveratrol demonstrated enhanced memory retention and improved functional connectivity in the hippocampus, a critical area for memory processing [56,59]. A long-term study (52 weeks) indicated that resveratrol treatment in individuals with mild-to-moderate Alzheimer’s disease led to a decline in amyloid-beta (Aβ) levels in cerebrospinal fluid, suggesting a potential role in mitigating Alzheimer’s pathology [56,60]. Despite some positive findings, numerous clinical trials report no significant improvements in cognitive function from resveratrol supplementation. A meta-analysis involving 225 patients found no significant effects on memory or cognitive performance, although it noted some improvements in mood parameters like vigor and fatigue [61]. Similarly, a study on individuals with schizophrenia showed no improvements in various cognitive tasks after one month of 200 mg/day resveratrol supplementation [62]. Another study involving 60 healthy adults assessed the impact of 500 mg/day resveratrol supplementation over 28 days. The results indicated that participants did not experience clear improvements in cognitive function despite some physiological changes, such as increased cerebral blood flow during cognitive tasks [63]. Thus, the effectiveness of resveratrol appears to vary significantly among different populations: while some studies indicate benefits for older adults or those with specific health conditions, others find no effects in younger or healthier individuals. This inconsistency suggests that factors such as age, health status, and dosage may influence the outcomes.

Resveratrol’s effects on cognition may be mediated through sirtuin activation and cerebral blood flow improvement. Resveratrol activates sirtuin proteins, particularly SIRT1, which plays a role in enhancing neuronal survival and synaptic plasticity, and improving glucose metabolism in the brain [26,59]. Resveratrol (10–500 mg/day) has been shown to improve cerebral blood flow and vascular function, which are essential for maintaining cognitive health, especially in aging populations [57,58,64]. The evidence suggests that resveratrol may enhance cognitive function, particularly in older adults and those at risk for neurodegenerative diseases. Its neuroprotective properties, combined with its ability to improve cerebrovascular function and activate beneficial cellular pathways, position resveratrol as a promising candidate for cognitive enhancement. However, further research is necessary to fully understand its efficacy and mechanisms in diverse populations.

### 2.3. Neuroprotection

Resveratrol exhibits strong antioxidant properties, which help mitigate oxidative stress—a significant factor in neuronal damage and cognitive decline. It scavenges free radicals and enhances the activity of endogenous antioxidant enzymes, thereby protecting neurons from oxidative injury [56,65]. Resveratrol reduces the expression of pro-inflammatory cytokines and inhibits the activation of NF-κB, a key regulator of inflammation. This action helps to lower neuroinflammation, which is often implicated in neurodegenerative diseases [56,66]. Resveratrol activates sirtuin 1 (SIRT1), a protein that plays a crucial role in cellular stress resistance, metabolism, and longevity. SIRT1 activation leads to deacetylation of various substrates involved in cell survival and synaptic plasticity, promoting neuronal health and function [66,67].

Resveratrol has been shown to inhibit the aggregation of Aβ peptides, which are central to the pathology of Alzheimer’s disease. By reducing Aβ levels and promoting its clearance, resveratrol may help protect against the neurotoxic effects associated with its accumulation [66,68]. However, research using animal models has shown mixed results regarding resveratrol’s ability to prevent Aβ aggregation. For instance, some studies indicated that resveratrol did not significantly inhibit Aβ formation or affect the activity of β- and γ-secretases, suggesting limitations in its neuroprotective potential against Alzheimer’s disease [69].

Resveratrol has been reported to restore the integrity of the blood–brain barrier (BBB) by decreasing levels of matrix metalloproteinases, which can disrupt BBB function. This restoration is crucial for maintaining brain health and preventing neuroinflammation [69]. While some studies report neuroprotective effects of resveratrol in models of ischemic stroke and neurodegeneration, others have failed to replicate these findings consistently. For example, certain experiments have shown no significant improvement in behavioral outcomes or reductions in infarct volume when comparing resveratrol-treated groups to controls [65,70].

Clinical trials have indicated that resveratrol supplementation can improve cognitive function and enhance cerebral blood flow in older adults. A study involving postmenopausal women demonstrated significant improvements in cognitive tests and perceived performance after resveratrol treatment [56,58]. In various animal studies, resveratrol has shown protective effects against cognitive decline induced by models of neurodegeneration. For instance, in models of Alzheimer’s disease, resveratrol administration improved learning and memory abilities, reduced oxidative stress markers, and enhanced neurogenesis in the hippocampus [65,66,68]. The evidence suggests that resveratrol has significant neuroprotective effects through multiple mechanisms, including antioxidant and anti-inflammatory actions, SIRT1 activation, and modulation of amyloid-beta aggregation. These properties contribute to its potential as a therapeutic agent for neurodegenerative diseases, particularly Alzheimer’s disease. Further research, including larger clinical trials, is needed to fully establish its efficacy and optimal dosing strategies for cognitive enhancement and neuroprotection.

### 2.4. Reproductive Health and Lifespan Expansion

Resveratrol acts as a potent antioxidant, reducing oxidative stress, which is crucial for maintaining ovarian function and overall reproductive health. This is particularly important in conditions like polycystic ovary syndrome (PCOS) and endometriosis, where oxidative stress is elevated [71,72]. It activates SIRT1, a protein associated with improved mitochondrial function and protection against oxidative damage in ovarian cells. This activation can enhance ovarian function and improve outcomes in assisted reproductive technologies (ART) like in vitro fertilization (IVF) [72,73]. Resveratrol exhibits phytoestrogenic properties, which may help in modulating hormonal balance and improving conditions related to reproductive health, such as menstrual disorders and infertility [71]. A study indicated that resveratrol supplementation improved ovarian sensitivity to exogenous follicle-stimulating hormone (FSH) in women over 35 years of age, suggesting potential benefits in IVF outcomes [73].

Resveratrol has shown promise in improving ovarian function and reducing symptoms in women with PCOS, potentially enhancing fertility outcomes in this population [3,74]. However, while some studies report improvements in oocyte quality and maturation, others have shown no significant differences in clinical pregnancy rates, highlighting the need for further research [74,75]. For instance, one study found that 200 mg/day resveratrol intake was associated with a decreased clinical pregnancy rate and an increased risk of miscarriage among women undergoing IVF [76]. In other randomized controlled trials involving women with polycystic ovary syndrome (PCOS), 800 mg/day resveratrol supplementation for 40 days before ovarian stimulation did not show significant differences in the number of mature oocytes, cleavage rate, fertilization rate, or overall fertility rates compared to the control group. Although there were improvements in high-quality oocyte and embryo rates, the primary fertility outcomes did not demonstrate significant benefits from resveratrol [73,76]. In studies examining hormonal changes associated with resveratrol supplementation, some findings indicated that 800 mg/day resveratrol did not significantly affect levels of follicle-stimulating hormone (FSH) or luteinizing hormone in certain populations. For instance, a pooled analysis revealed no significant effect of resveratrol on FSH levels, suggesting limited impact on key hormones involved in reproductive health [77]. Furthermore, research involving animal models has shown that high doses of resveratrol can disrupt normal ovarian function and hormonal balance, potentially leading to negative reproductive outcomes. For example, studies demonstrated that high doses could block estrogen receptors and disrupt estrous cycles in female rats, raising concerns about its safety and efficacy in enhancing reproductive health [71].

Resveratrol is associated with lifespan extension through several biological pathways. Resveratrol activates sirtuins, particularly SIRT1, which is linked to the effects of caloric restriction—known to extend lifespan in various organisms. This activation promotes cellular stress resistance and metabolic health [57,71]. By reducing chronic inflammation and oxidative stress, resveratrol may mitigate age-related diseases, thereby contributing to increased lifespan [71].

Resveratrol has been shown to improve cognitive function in postmenopausal women and in older adults, suggesting its potential role in extending healthy lifespan through enhanced brain health and potentially slowing cognitive decline associated with aging [57]. Resveratrol’s antioxidant and anti-inflammatory properties contribute to its potential in reducing the risk of chronic diseases such as cardiovascular disease and diabetes, which are significant factors in longevity [71]. However, an Italian study found no association between resveratrol levels in the diet and reduced incidence of cardiovascular disease, cancer, or overall mortality [78]. Other clinical trials investigating the effects of resveratrol on healthy individuals and those with age-related conditions have yielded contradictory outcomes regarding its impact on longevity. Some trials reported no significant improvements in health status or lifespan among participants consuming resveratrol supplements, reinforcing the notion that more research is needed to clarify its role in human aging [1]. Such conflicting results correlate with findings in mice: while resveratrol (0.03% and 0.12% in the diet) can activate longevity-related pathways in rodents, it does not consistently extend lifespan, and genetically heterogeneous mice on a standard fat diet did not experience lifespan extension with resveratrol supplementation at certain concentrations. This suggests that dietary context and genetic background significantly influence the effectiveness of resveratrol for lifespan extension [79].

Thus, resveratrol shows promise in both reproductive health and lifespan expansion through its antioxidant properties, modulation of hormonal balance, and activation of beneficial cellular pathways. While preliminary findings are encouraging, further research is needed to fully understand its efficacy and mechanisms in diverse populations, particularly regarding its use in clinical settings for reproductive health and aging.

## 3. Effects and Mechanisms of Extra Virgin Olive Oil

### 3.1. Molecular Mechanism of EVOO Components Activity

**Figure 1:**
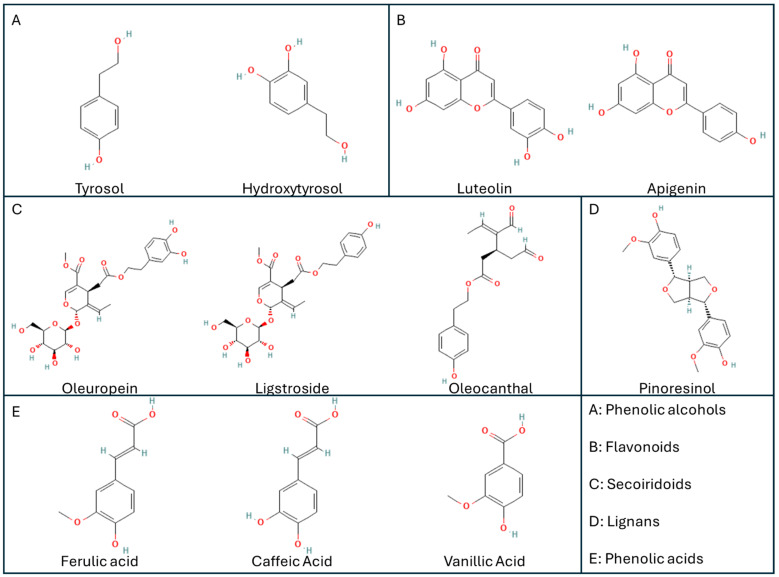
EVOOs phenolic compounds.

EVOO is widely recognized for its health benefits, attributed to its unique composition of fatty acids, antioxidants, and bioactive compounds. This discourse evaluates the effects and mechanisms of EVOO, particularly focusing on its cardiovascular, anti-inflammatory, and neuroprotective properties. EVOO is rich in oleic acid, a monounsaturated fatty acid that has been shown to reduce bad cholesterol levels (LDL) while increasing good cholesterol (HDL). This lipid profile is associated with a lower risk of heart disease [80]. EVOO contains many bioactive compounds, which could not be obtained via chemical synthesis, e.g., phenolic compounds such as hydroxytyrosol, tyrosol, as well as the secoiridoids oleacein and oleocanthal (Figure 1), playing central roles as anti-inflammatory, neuro-protective, and anti-cancer agents [81,82].

The phenolic compounds in EVOO, such as hydroxytyrosol and oleuropein, exhibit strong antioxidant properties. They help prevent the oxidation of LDL cholesterol, a key factor in the development of atherosclerosis and cardiovascular diseases [83]. EVOO has been shown to modulate inflammatory markers. The phenolic compounds can inhibit the expression of pro-inflammatory cytokines and enzymes, such as cyclooxygenase-2 (COX-2) and lipoxygenase, which play a role in chronic inflammation linked to various diseases, including arthritis and metabolic syndrome [83,84].

The Mediterranean diet, which includes high consumption of EVOO, has been associated with lower levels of systemic inflammation, contributing to overall health and longevity [15]. EVOO’s antioxidant capacity helps protect neurons from oxidative damage, which is crucial in preventing neurodegenerative diseases like Alzheimer’s and Parkinson’s. Studies have indicated that EVOO can enhance cognitive function and reduce the risk of cognitive decline in aging populations [84].

The activation of SIRT1 by EVOO’s polyphenols is linked to improved neuronal health and longevity. SIRT1 activation promotes mitochondrial function and reduces neuroinflammation, which are vital for maintaining cognitive health as one ages [83,84]. The health benefits of EVOO are largely attributed to its bioactive compounds, particularly polyphenols. Hydroxytyrosol, one of the most potent antioxidants in EVOO, has been shown to induce apoptosis in cancer cells and reduce the expression of adhesion molecules on endothelial cells, thus preventing cardiovascular diseases [83].

Other phenolic compounds contribute to the overall antioxidant capacity of EVOO, enhancing its protective effects against oxidative stress and inflammation [83,84]. EVOO is a critical component of a healthy diet, particularly the Mediterranean diet, due to its numerous health benefits [18]. Its effects on cardiovascular health, anti-inflammatory properties, and neuroprotection are primarily mediated through its rich composition of monounsaturated fatty acids and bioactive compounds [12,13,14,15]. Understanding these mechanisms can help individuals make informed dietary choices to enhance their overall health and longevity.

The molecular mechanisms of the bioactive components in EVOO are crucial for understanding its health benefits. EVOO is rich in monounsaturated fatty acids and various phenolic compounds, which contribute to its antioxidant, anti-inflammatory, and neuroprotective properties. The primary fatty acid in EVOO—oleic acid—plays a significant role in cardiovascular health by improving lipid profiles, lowering LDL cholesterol, and raising HDL cholesterol. This lipid composition is vital for maintaining cellular membrane integrity and fluidity, which is essential for proper cell function. The phenolic compounds are among the most studied components of EVOO. They exhibit strong antioxidant properties, which help neutralize free radicals and reduce oxidative stress. Hydroxytyrosol, in particular, has been shown to activate the MAPK and mammalian target of rapamycin (mTOR) pathways, contributing to cellular homeostasis and longevity [84].

The antioxidant activity of phenolic compounds is primarily due to their ability to donate hydrogen atoms to free radicals, thus terminating the chain reaction of lipid oxidation. This action stabilizes the phenoxyl radicals formed during the reaction, enhancing the overall antioxidant capacity of EVOO [85]. The phenolic compounds in EVOO act as chain-breaking antioxidants. They inhibit the oxidation of lipids and other biomolecules, which is crucial for preventing chronic diseases associated with oxidative stress. The presence of hydroxyl groups in these compounds enhances their ability to scavenge free radicals [85,86]. EVOO consumption has been associated with reduced levels of inflammatory markers. The phenolic compounds can inhibit the expression of pro-inflammatory cytokines and enzymes, such as COX-2, thereby mitigating chronic inflammation [84]. Furthermore, oleocanthal shares a chemical structure with non-steroidal anti-inflammatory drugs, such as ibuprofen, which allows us to hypothesize that EVOO compounds could directly inhibit COX-2 [87].

The neuroprotective effects of EVOO components, particularly its phenolic compounds, are linked to their ability to reduce oxidative stress and inflammation in the brain. Studies have shown that these compounds can improve cognitive function and protect against neurodegenerative diseases by modulating pathways involved in neuronal survival and apoptosis [65,66]. EVOO has been shown to enhance blood circulation and reduce platelet aggregation, which is beneficial for cardiovascular health. This effect is attributed to the minor components of EVOO that influence coagulation factors and improve endothelial function [84].

The molecular mechanisms of EVOO components, particularly its monounsaturated fatty acids and phenolic compounds, play a significant role in its health benefits. Through their antioxidant, anti-inflammatory, and neuroprotective properties, these components contribute to the prevention of chronic diseases and promote overall health. Continued research is essential to fully elucidate the complex interactions and health benefits associated with EVOO consumption.

EVOO plays a significant role in mitigating age-related diseases and enhancing cognitive function, particularly through its rich composition of monounsaturated fats and bioactive compounds, including phenolic compounds. Effects of EVOO on age-related diseases include improvements in mild cognitive impairment (MCI). Recent studies indicate that daily consumption of EVOO can improve cognitive function in individuals with mild cognitive impairment. A study conducted by Amal Kaddoumi at Auburn University found that participants consuming EVOO showed enhanced brain connectivity and reduced blood–brain barrier permeability, which are critical factors in maintaining cognitive health [88].

EVOO consumption has been linked to alterations in biomarkers related to Alzheimer’s disease, such as beta-amyloid and tau phosphorylation. These changes suggest that EVOO may influence the processing and clearance of these neurotoxic proteins, potentially reducing the risk of Alzheimer’s progression [88].

EVOO is rich in antioxidants, particularly phenolic compounds like hydroxytyrosol and oleocanthal, which help combat oxidative stress and inflammation—two key factors in the aging process and the development of neurodegenerative diseases. These compounds can modulate inflammatory pathways and enhance antioxidant defenses in the brain, contributing to improved cognitive function and reduced risk of age-related diseases [84,87,88].

The consumption of EVOO is associated with improved cardiovascular health, which is closely linked to cognitive function. The monounsaturated fats in EVOO help lower LDL cholesterol and improve HDL cholesterol levels, reducing the risk of cardiovascular diseases that can exacerbate cognitive decline. Additionally, the anti-inflammatory properties of EVOO contribute to better vascular health, ensuring adequate blood flow to the brain [88].

The health benefits of EVOO are largely attributed to its bioactive compounds. Phenolic compounds exhibit strong antioxidant and anti-inflammatory effects, protecting neurons from oxidative damage and enhancing synaptic plasticity. Hydroxytyrosol, for instance, has been shown to improve neuron function and reduce neuroinflammation [82,84,85]. Oleic acid is known for its cardioprotective properties and may also play a role in neuroprotection by promoting neurogenesis and improving overall brain health [88]. EVOO consumption has been shown to enhance the integrity of the BBB, which is crucial for preventing neurotoxic substances from entering the brain. Improved BBB function can lead to better cognitive outcomes and reduced risk of neurodegenerative diseases [88].

EVOO activates various neuroprotective pathways. The activation of SIRT1 by EVOO’s polyphenols promotes cellular stress resistance and metabolic health, which are essential for maintaining cognitive function as one ages [82,84,85]. EVOO has been associated with reduced levels of Aβ, a hallmark of Alzheimer’s disease, thereby potentially slowing the progression of cognitive decline [88].

Thus, EVOO serves as a powerful ally in combating age-related diseases, particularly cognitive decline. Its rich composition of monounsaturated fats and bioactive compounds contributes to improved brain health, enhanced cognitive function, and reduced risk of neurodegenerative diseases. The mechanisms by which EVOO exerts these effects include antioxidant and anti-inflammatory actions, enhancement of blood–brain barrier integrity, and modulation of key neuroprotective pathways. Future research should continue to explore the long-term effects of EVOO consumption on cognitive health and its potential role in preventing age-related diseases.

### 3.2. EVOO Contribution to Cognitive Enhancement

EVOO has been increasingly recognized for its potential to enhance cognitive function and mitigate age-related cognitive decline. EVOO is a staple of the Mediterranean diet, known for its high content of monounsaturated fatty acids and bioactive compounds, particularly phenolic compounds [12,13,14,15]. Research suggests that regular consumption of EVOO is associated with improved cognitive performance and a reduced risk of neurodegenerative diseases, including Alzheimer’s disease. EVOO is rich in phenolic compounds like oleuropein and hydroxytyrosol, which have demonstrated antioxidant and anti-inflammatory properties. These compounds help protect neurons from oxidative stress and inflammation, both of which are critical factors in cognitive decline and neurodegeneration [89,90].

A study involving participants with MCI found that daily consumption of 30 mL of EVOO significantly improved clinical dementia ratings and behavioral scores. Participants who consumed EVOO showed enhanced functional connectivity in the brain and reduced permeability of the blood–brain barrier (BBB), which is often compromised in neurodegenerative conditions [88]. The PREDIMED-NAVARRA trial demonstrated that participants following a Mediterranean diet supplemented with EVOO (1 L per week) scored higher on cognitive tests compared to those on a low-fat diet. This suggests that the incorporation of EVOO into a balanced diet can lead to sustained cognitive benefits over time [89]. However, a systematic review of various studies on olive oil consumption and cognitive performance indicated inconsistencies in findings. While some studies reported positive associations between EVOO intake and cognitive health, others did not find significant improvements across various cognitive domains. This inconsistency suggests that while EVOO may have potential benefits, it does not uniformly enhance cognitive function in all populations [89]. While EVOO may improve cognitive function in older adults without dementia, its effects are less clear in younger or healthier individuals. Some studies indicate no significant neuroprotective benefits when examining populations outside the high-risk groups for cognitive decline, underscoring the variability of EVOO’s effectiveness based on demographic factors [89,90].

Regular EVOO consumption has been linked to a lower risk of developing Alzheimer’s disease and other forms of dementia. Studies indicate that a high intake of EVOO is associated with better memory and cognitive function in older adults [90]. EVOO has been shown to enhance the integrity of the blood–brain barrier, which protects the brain from neurotoxins and facilitates the clearance of waste products. Improved BBB function is crucial for maintaining cognitive health and preventing neurodegeneration [88].

EVOO consumption has been associated with reduced levels of amyloid-beta and tau proteins, which are hallmarks of Alzheimer’s disease. The alteration in the processing and clearance of these proteins may contribute to the neuroprotective effects of EVOO [88]. The evidence supporting the cognitive enhancement effects of EVOO is compelling. Its rich composition of monounsaturated fats and bioactive compounds provides significant neuroprotective benefits, particularly in the context of aging and cognitive decline. Incorporating EVOO into a balanced diet, especially as part of the Mediterranean diet, may not only improve cognitive function but also reduce the risk of neurodegenerative diseases [20].

While EVOO is generally regarded as beneficial for brain health, it was shown that refined olive oil (ROO), which lacks phenolic compounds, can also produce positive effects on cognitive function. The study did not find distinct neuroprotective effects attributable specifically to EVOO over ROO. Although EVOO is richer in phenolic compounds, the improvements observed in the ROO group suggest that oleic acid alone may also contribute to cognitive benefits, indicating that EVOO may not have unique neuroprotective properties. This raises questions about the necessity of phenolic compounds for achieving cognitive benefits and suggests that other components of olive oil, such as oleic acid, may also contribute positively to brain health [88]. Thus, further research is warranted to explore the long-term effects and underlying mechanisms of EVOO on brain health.

### 3.3. Neuroprotection

EVOO has garnered significant attention for its neuroprotective effects, particularly in relation to cognitive decline and neurodegenerative diseases such as Alzheimer’s disease. EVOO contains a high concentration of monounsaturated fatty acids, primarily oleic acid, and various phenolic compounds, including oleuropein and hydroxytyrosol. These components are known for their antioxidant and anti-inflammatory properties, which are crucial for protecting neuronal health. The phenolic compounds in EVOO help combat oxidative stress by scavenging free radicals and enhancing the body’s antioxidant defenses. This action reduces oxidative damage to neurons, which is a significant factor in the progression of neurodegenerative diseases. EVOO has been shown to inhibit the activation of pro-inflammatory pathways. By reducing the activation of astrocytes and microglia, which are involved in neuroinflammation, EVOO helps mitigate the inflammatory responses that can lead to neuronal damage and cognitive decline [91,92,93].

Research indicates that EVOO enhances the clearance of Aβ peptides from the brain. A study using transgenic mouse models of Alzheimer’s disease demonstrated that long-term consumption of an EVOO-enriched diet significantly reduced Aβ levels and tau pathology, which are critical hallmarks of Alzheimer’s disease. This reduction is attributed to improved clearance mechanisms and modulation of amyloid precursor protein processing [94,95]. However, a cohort study found that high olive oil intake did not provide protective effects against Alzheimer’s disease or memory decline. The researchers concluded that the primary fatty acids in olive oil, such as oleic acid, linoleic acid, and palmitic acid, do not contribute significantly to cognitive functioning compared to omega-3 fatty acids found in fish oil. This study highlights the limitations of relying solely on olive oil for cognitive health benefits, particularly in populations at risk for dementia [89].

EVOO has been shown to enhance the integrity and functionality of the BBB. Compromised BBB is often observed in neurodegenerative diseases, leading to increased permeability and the entry of neurotoxic substances into the brain. Studies have demonstrated that EVOO consumption can reduce BBB permeability, thereby protecting the brain from potential damage [88]. Clinical studies have reported that individuals consuming EVOO as part of a Mediterranean diet exhibit better cognitive performance and a reduced risk of developing MCI or dementia. For instance, a randomized controlled trial found that participants who included EVOO in their diet showed significant improvements in clinical dementia ratings and cognitive function compared to those consuming refined olive oil [88]. In a large population-based cross-sectional study population, greater adherence to such diets resulted in better cognitive function and lower risk of cognitive impairment, and the association was independent [96].

The neuroprotective effects of EVOO are mediated through several mechanisms. EVOO promotes autophagy, a cellular process that helps clear damaged proteins and organelles, thereby reducing the accumulation of toxic substances such as Aβ. EVOO may enhance the expression of neurotrophic factors like brain-derived neurotrophic factor (BDNF), which supports neuronal survival, growth, and synaptic plasticity. The neuroprotective effects of EVOO are supported by a growing body of research highlighting its role in reducing oxidative stress, inflammation, and amyloid-beta accumulation, as well as enhancing blood–brain barrier integrity [88]. These mechanisms contribute to improved cognitive function and a lower risk of neurodegenerative diseases. Incorporating EVOO into the diet, particularly as part of a Mediterranean dietary pattern, may offer significant benefits for brain health and cognitive enhancement, especially in aging populations [12,13,14,15]. Further research, particularly in human clinical trials, is essential to fully elucidate the extent of these benefits and the underlying mechanisms involved.

The scientific research dedicated to the neuroprotective properties of EVOO has expanded significantly in recent years, revealing promising results regarding its potential to enhance cognitive function and mitigate the effects of neurodegenerative diseases. Research has demonstrated that EVOO consumption can improve synaptic activity, memory, and cognitive performance in animal models. For example, studies in mice showed that EVOO reduced tau neuropathology and improved short-term plasticity, suggesting a protective effect against cognitive decline associated with aging and Alzheimer’s disease [89,90].

Clinical trials, such as the PREDIMED-NAVARRA study, indicated that individuals consuming a Mediterranean diet enriched with EVOO exhibited better cognitive functioning compared to those on a control diet. Participants in the EVOO group showed reduced rates of MCI and improved scores on cognitive assessments [89,90].

The phenolic compounds in EVOO, such as oleuropein and hydroxytyrosol, possess strong antioxidant properties that protect neurons from oxidative stress. These compounds also exhibit anti-inflammatory effects by inhibiting pro-inflammatory cytokines, which can contribute to neurodegenerative processes [90]. EVOO has been shown to enhance the integrity of the BBB, which is crucial for maintaining brain health. A study involving participants with MCI found that daily consumption of EVOO reduced BBB permeability and improved functional connectivity in the brain. This is significant, as a compromised BBB is often associated with the onset of neurodegenerative diseases [88].

EVOO consumption has been linked to alterations in biomarkers associated with Alzheimer’s disease. In studies, EVOO was found to reduce levels of amyloid-beta (Aβ) and phosphorylated tau proteins, suggesting that it may modulate the processing and clearance of these neurotoxic substances [88]. This effect was observed in both animal models and human trials, supporting the potential of EVOO in delaying the progression of Alzheimer’s disease.

Several randomized controlled trials have been conducted to assess the effects of EVOO on cognitive function. For instance, a trial involving 26 participants with MCI showed significant improvements in clinical dementia ratings and behavioral scores after six months of EVOO consumption [88]. The study highlighted the need for further research to confirm these findings and explore the mechanisms involved. Long-term studies have indicated that higher olive oil intake is associated with a lower risk of cognitive decline and dementia. The evidence suggests that regular consumption of EVOO as part of a balanced diet may contribute to better cognitive health in aging populations [89,90]. The body of scientific research indicates that EVOO has significant neuroprotective properties, primarily through its antioxidant and anti-inflammatory effects, enhancement of blood–brain barrier integrity, and modulation of Alzheimer’s disease biomarkers. While the findings are promising, further research, particularly large-scale clinical trials, is necessary to fully understand the mechanisms of action and the long-term benefits of EVOO on cognitive health. Current evidence supports the inclusion of EVOO in dietary patterns aimed at promoting brain health and preventing neurodegenerative diseases.

### 3.4. Reproductive Health and Lifespan Expansion

Many studies that explore the health benefits of EVOO often focus on cardiovascular or metabolic outcomes rather than reproductive health specifically. As a result, there may be insufficient data to draw firm conclusions about its efficacy in improving reproductive health outcomes. While the research on the direct effects of EVOO on reproductive health is limited, there are a few key ways it may provide benefits. A study found that men whose diets were higher in monounsaturated fats, like those found in olive oil, had a higher proportion of mature sperm and fewer abnormal sperm compared to men with lower intakes [97]. The antioxidants in EVOO may also help protect sperm from oxidative damage. Some research suggests a Mediterranean diet rich in EVOO may increase pregnancy rates in women undergoing IVF by up to 40% [98]. However, more research is needed to confirm a direct link between EVOO consumption and fertility in women. A Mediterranean diet with olive oil may offer some protection against developing gestational diabetes during pregnancy [99]. Gestational diabetes can lead to complications for both mother and baby if not properly managed. Several studies have linked high olive oil intake to maintaining healthy bones, including reduced risk of hip fracture and improved bone density [100]. This may be especially important for women during and after menopause when bone loss accelerates. In summary, while the evidence is not conclusive, the anti-inflammatory properties, healthy fats, and antioxidants in EVOO may provide some benefits for male fertility, female fertility, pregnancy health, and bone health in women. Incorporating EVOO into an overall healthy diet may be a simple way to potentially support reproductive health.

Numerous studies have found a strong correlation between the regular consumption of EVOO and increased lifespan. A Harvard study that pooled data from over 92,000 healthy men and women found that those who consumed the most olive oil (over 1/2 tablespoon per day) had a 19% lower risk of death from any cause over a 28-year period, compared to those who rarely or never consumed olive oil. Replacing other fats like butter, margarine, or mayonnaise with olive oil was associated with up to a 34% lower mortality rate [101]. Higher olive oil intake was linked to lower risks of death from cardiovascular disease, cancer, neurodegenerative disease, and lung disease [101]. A Mediterranean diet rich in EVOO has been associated with reduced incidence of age-related cognitive decline, Parkinson’s disease, Alzheimer’s disease, cardiovascular diseases, cancer, and diabetes [16]. EVOO has been shown to modulate the “hallmarks of aging”—the key cellular and molecular mechanisms that drive the aging process. This includes reducing oxidative stress, inflammation, genomic instability, epigenetic alterations, and cellular senescence [16]. The monounsaturated fatty acids and polyphenol compounds in EVOO, particularly secoiridoids like oleuropein and oleocanthal, are thought to underlie these anti-aging effects [19,87]. In summary, the consistent evidence from observational studies, animal models, and cellular experiments indicates that the regular consumption of EVOO, as part of an overall healthy diet and lifestyle, may be an effective strategy to promote healthy aging and longevity. The unique fatty acid profile and abundance of antioxidant polyphenols in EVOO appear to be key factors in its anti-aging effects.

The correlation between EVOO consumption and increased lifespan is supported by a range of studies highlighting its health benefits, particularly in relation to aging. Numerous studies have established that adherence to a Mediterranean diet, which prominently features EVOO, is associated with lower mortality rates and increased longevity. For instance, a comprehensive review indicated that EVOO has a preventive role against common age-related diseases such as cardiovascular and neurodegenerative disorders, cancer, and diabetes [16]. EVOO is rich in polyphenols that modulate cellular pathways related to oxidative stress and inflammation, which are pivotal in the aging process. Research has shown that these compounds can activate protective cellular mechanisms, such as the Nrf-2 pathway, and inhibit pro-inflammatory pathways like NF-κB, thereby potentially slowing down the aging process and reducing the risk of age-related diseases [16,19].

Animal models have demonstrated that diets enriched with EVOO can improve health markers associated with aging. For example, studies involving SAMP8 mice, a model for accelerated aging, showed that EVOO reduced oxidative stress and improved cognitive functions compared to control diets [102]. These findings suggest that the phenolic compounds in EVOO can exert protective effects on brain health, which is crucial for maintaining quality of life as one ages. Epidemiological studies have consistently linked high olive oil consumption to a reduced risk of mortality. A notable study found that individuals consuming high amounts of olive oil had a 19% lower risk of death from cardiovascular diseases and a 17% lower risk of death from cancer over a 28-year period [101]. These findings underscore the potential of EVOO to contribute to longevity through its cardioprotective and anti-cancer properties. On the other hand, such assumptions should be made with caution, as a study involving male Wistar rats found that while dietary virgin olive oil decreased DNA double-strand breaks compared to sunflower oil, there were no significant differences in mean or maximum lifespan between the groups. This suggests that while EVOO may have protective effects against cellular damage, it does not necessarily translate into increased longevity [19].

The beneficial effects of EVOO are attributed to its high content of monounsaturated fats and bioactive compounds, particularly oleocanthal and hydroxytyrosol, which have been shown to possess anti-inflammatory and antioxidant properties. These compounds help control oxidative stress, a major contributor to aging and chronic diseases [101,103]. The body of evidence supporting the correlation between EVOO consumption and increased lifespan is substantial. The combination of epidemiological studies, animal research, and mechanistic insights into how EVOO impacts cellular health points to its significant role in promoting longevity and reducing the incidence of age-related diseases. However, it should be noted that in studies assessing the impact of diets rich in EVOO on fertility and reproductive health, confounding factors such as overall dietary patterns, lifestyle choices, and individual health conditions can complicate the interpretation of results. This variability may lead to inconclusive findings regarding the direct benefits of EVOO alone. Continued research is necessary to further elucidate the specific mechanisms and to confirm these effects in diverse human populations.

## 4. Comparison Between Resveratrol and Extra Virgin Olive Oil

### 4.1. Comparable Effects

Resveratrol and EVOO are both celebrated for their health benefits, particularly in the context of aging and age-related diseases. This discussion highlights their shared protective effects, mechanisms of action, and the scientific evidence supporting their roles in promoting health and longevity. Both resveratrol and EVOO are rich in antioxidants, which play a crucial role in combating oxidative stress—a significant contributor to aging and various chronic diseases. This polyphenolic compound is known for its potent antioxidant activity, which helps neutralize free radicals and reduce oxidative damage to cells. Studies have shown that resveratrol can enhance the body’s natural antioxidant defenses, leading to improved cellular health and longevity [1,29]. Particularly high in phenolic compounds such as hydroxytyrosol and oleuropein, EVOO also exhibits strong antioxidant properties. These compounds help protect against oxidative stress by scavenging free radicals and reducing inflammation, which are vital for maintaining cellular integrity and function [82,84,85].

Chronic inflammation is a common underlying factor in many age-related diseases, including cardiovascular disease, diabetes, and neurodegenerative disorders. It has been shown to reduce inflammation by inhibiting pro-inflammatory cytokines and pathways, such as the NF-κB signaling pathway. This anti-inflammatory action contributes to its protective effects against various chronic diseases [29]. The phenolic compounds in EVOO also exert anti-inflammatory effects by modulating inflammatory pathways. Regular consumption of EVOO is associated with lower levels of inflammatory markers in the body, which can help reduce the risk of chronic diseases [16,82,84,85].

**Figure 2:**
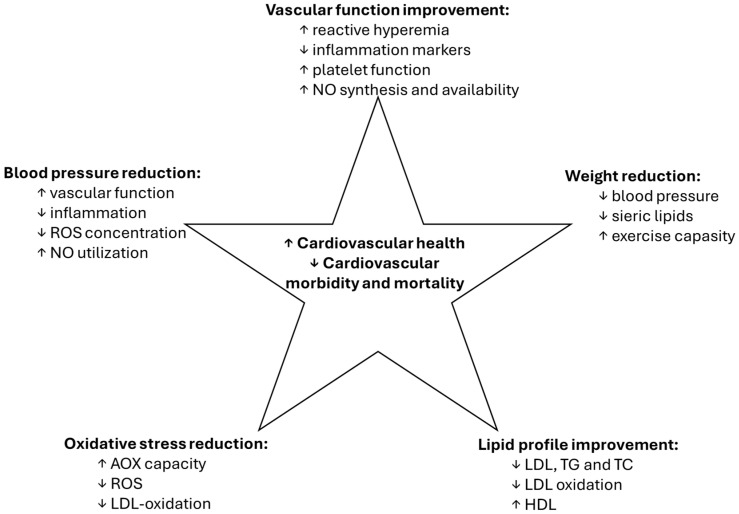
Shared effects of resveratrol and EVOO. Note: EVOO—extra virgin olive oil; ROS—reactive oxygen species; NO—nitric oxide; LDL—low density lipoproteins; TG—triglycerides; TC—total cholesterol; AOX—antioxidant; ↑—improvement; ↓—decrease.

Both resveratrol and EVOO have demonstrated significant cardiovascular benefits (Figure 2), which are crucial for promoting longevity. Research indicates that resveratrol can improve endothelial function, reduce blood pressure, and lower LDL cholesterol levels, contributing to a decreased risk of cardiovascular diseases [1]. Its ability to enhance nitric oxide production also plays a role in vasodilation and improved blood flow. The monounsaturated fats in EVOO, particularly oleic acid, are known to improve lipid profiles by increasing HDL cholesterol and decreasing LDL cholesterol. Studies show that diets rich in EVOO are linked to lower rates of heart disease and improved cardiovascular health [16,80,82,84,85].

Both compounds have been associated with cognitive health and may help protect against neurodegenerative diseases. It has been shown to enhance cognitive function and protect against neurodegeneration by reducing oxidative stress and inflammation in the brain. Resveratrol’s ability to activate SIRT1, a protein associated with longevity, further supports its neuroprotective properties [1,29]. The consumption of EVOO has been linked to a reduced risk of cognitive decline and Alzheimer’s disease. Studies suggest that the phenolic compounds in EVOO can help clear amyloid-beta plaques from the brain, which are associated with Alzheimer’s pathology [16,82,90].

Both resveratrol and EVOO exhibit properties that may help reduce the risk of certain cancers. Its antioxidant and anti-inflammatory effects contribute to its anti-cancer properties. Research suggests that resveratrol can inhibit cancer cell growth and promote apoptosis (programmed cell death) in various cancer types [1,29]. The polyphenols in EVOO have been associated with a reduced risk of certain cancers, particularly breast and digestive system cancers. The antioxidant properties of EVOO help protect cells from DNA damage and promote healthy cell function [82,84,85]. Resveratrol and EVOO share numerous health benefits that contribute to their roles as protective agents against age-related diseases. Their antioxidant and anti-inflammatory properties, cardiovascular protection, neuroprotective effects, and potential for cancer prevention highlight their significance in promoting health and longevity. Incorporating both resveratrol-rich foods (like grapes and red wine) and EVOO into a balanced diet may offer synergistic effects that enhance overall well-being and reduce the risk of chronic diseases associated with aging. Continued research is essential to fully understand their mechanisms and optimize their use in health promotion.

### 4.2. Potential Synergistic Interaction of Their Combination

**Figure 3:**
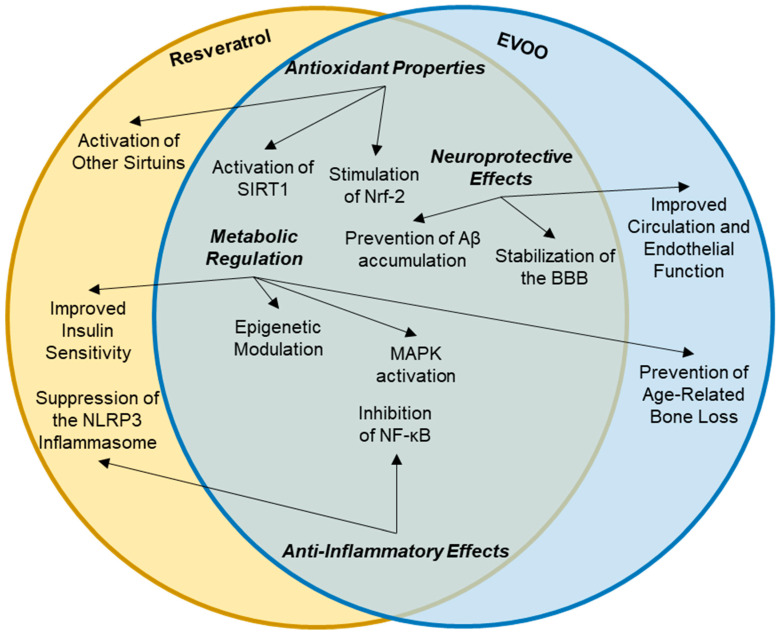
Molecular targets of resveratrol and EVOO. Note: EVOO—extra virgin olive oil; SIRT1—sirtuin 1; Nrf-2—nuclear factor erythroid 2-related factor 2; Aβ—amyloid-beta; BBB—blood–brain barrier; MAPK—mitogen-activated protein kinase; NLRP3—NLR family pyrin domain containing 3; NF-κB—nuclear factor κB.

The combination of resveratrol and EVOO presents a synergistic approach to enhancing health benefits, particularly in the context of age-related diseases, which is possible due to its cross-linking synergetic mechanisms of action (Figure 3). Both substances are rich in bioactive compounds that contribute to their protective effects against chronic diseases, including cardiovascular diseases, neurodegenerative disorders, and cancer. This discussion explores their unique advantages, pharmacokinetic and pharmacodynamic interactions, and the potential for combined therapeutic applications. Resveratrol has low bioavailability due to rapid metabolism and elimination from the body. Studies have shown that combining resveratrol with a fat matrix, such as that found in EVOO, can enhance its absorption and bioavailability. The fat content in EVOO can facilitate the intestinal absorption of resveratrol and its metabolites, leading to higher plasma concentrations and prolonged effects in the body [104].

The presence of EVOO may influence the metabolism of resveratrol, promoting the formation of beneficial metabolites. For instance, EVOO has been shown to increase the formation of glucuronide conjugates of resveratrol, which are more stable and may have enhanced biological activity compared to the parent compound [104]. Both resveratrol and EVOO possess strong antioxidant properties, which help combat oxidative stress and inflammation—key factors in the development of age-related diseases. Their combined effects may lead to a more significant reduction in oxidative damage and inflammatory responses, enhancing overall health outcomes [92,105].

Resveratrol is known for its cardioprotective effects, including improving endothelial function and reducing blood pressure. EVOO, rich in monounsaturated fats and polyphenols, also contributes to cardiovascular health by improving lipid profiles and reducing inflammation. Together, they may provide a robust protective effect against cardiovascular diseases [29,105]. Both compounds have been linked to cognitive health. Resveratrol has shown potential in improving memory and cognitive function, while EVOO has been associated with a reduced risk of neurodegenerative diseases. Their combination may enhance neuroprotective effects through synergistic mechanisms, such as reducing amyloid-beta accumulation and promoting neuronal health [29,92].

The presence of EVOO can enhance the absorption of resveratrol in the gastrointestinal tract. This is particularly important given resveratrol’s low bioavailability when taken alone. The lipid matrix provided by EVOO aids in the emulsification and absorption of resveratrol, leading to higher plasma levels and improved therapeutic effects [104]. Resveratrol undergoes extensive metabolism in the liver, primarily through conjugation with glucuronides and sulfates. The combination with EVOO may alter the metabolic pathway of resveratrol, potentially leading to a higher proportion of active metabolites in circulation. This could enhance the pharmacodynamic effects of resveratrol, making it more effective in exerting its protective benefits [11,106,107].

The combination of resveratrol and EVOO may lead to enhanced pharmacodynamic effects due to their complementary mechanisms. For instance, both compounds can activate similar signaling pathways, such as the SIRT1 pathway, which is involved in cellular stress resistance and longevity. This synergy may amplify their individual benefits, leading to improved health outcomes [29,92]. The combination of resveratrol and EVOO can be developed into functional food products aimed at promoting health and preventing age-related diseases. By leveraging their synergistic effects, such products could offer enhanced protective benefits compared to either component alone, making them appealing for health-conscious consumers [92,105]. Moreover, both EVOO and resveratrol are shown to be LINE-1 retrotransposon blockers. This blockade may help maintain genomic integrity by preventing excessive retrotransposition events, which can lead to mutations and cancer progression, and the inhibition of hypomethylation can potentially serve as a therapeutic strategy to stabilize the genome and reduce cancer risk [108,109].

Another critical aspect of cellular aging and cancer is telomere shortening, and resveratrol demonstrated pro-telomerase activity [110], while studies have shown that adherence to a diet rich in olive oil correlates with increased telomere length in certain populations, suggesting that dietary factors may influence telomere maintenance and overall cellular health [19]. Furthermore, both substances are involved in epigenetic regulation: resveratrol reduces DNA methyltransferase (DNMT) activity and their mRNA levels in breast cancer cells, leading to changes in DNA methylation, and may modulate histone deacetylase (HDAC) activity [111], while EVOO could affect miRNA expression, inhibiting DNMT activity and expression [112].

The combination of resveratrol and EVOO presents unique advantages based on their synergism (see Table 1). Enhanced bioavailability, complementary mechanisms of action, and synergistic pharmacokinetic and pharmacodynamic interactions contribute to their potential as protective agents. Further research is needed to explore the optimal formulations and dosages for maximizing their health benefits, as well as to clarify the underlying mechanisms involved. This combination holds promise for developing effective dietary strategies to improve overall health and longevity.

### 4.3. Advances in Development of Delivery System for Resveratrol

Despite resveratrol’s promising therapeutic potential, its clinical application has been limited by several key challenges. Resveratrol has poor water solubility, which reduces its bioavailability and makes formulation difficult [42,113]. Resveratrol undergoes rapid metabolism in the intestine and liver, primarily through conjugation to sulfate and glucuronide metabolites. This results in low oral bioavailability, often less than 1% [2,10].

Resveratrol is sensitive to oxidation and photosensitivity, further limiting its stability and bioavailability [42]. To overcome these limitations, various nanoparticle-based delivery systems have been developed to enhance resveratrol’s solubility, stability, and bioavailability [10,11]. Resveratrol can be encapsulated in biodegradable polymers like PLGA to improve its aqueous solubility and stability. These nanoparticles also provide controlled release of the drug [42,113]. Resveratrol can be incorporated into the lipid bilayer of liposomes, which protects it from degradation and enhances absorption. Liposomal formulations have shown improved bioavailability compared to free resveratrol [38,42]. Solid lipid nanoparticles (SLNs) are colloidal carriers made from lipids that are solid at room temperature. They can solubilize resveratrol and provide controlled release. SLNs have demonstrated enhanced brain delivery and neuroprotective effects in animal studies [42,106].

Resveratrol can be incorporated into the hydrophobic core of polymeric micelles, improving its solubility and stability. Crosslinked micelles provide additional stability in vivo [113]. Resveratrol has been combined with various chemotherapeutic drugs like oxaliplatin, cisplatin, 5-FU, and doxorubicin to enhance its anti-cancer effects. The combination therapy shows promising results in preclinical studies [10].

Combining resveratrol with EVOO may provide additional benefits. The fat matrix provided by EVOO may enhance the absorption and bioavailability of resveratrol. Studies show that the combination of resveratrol with EVOO increases the urinary recovery of resveratrol metabolites compared to resveratrol alone [104,106]. EVOO is rich in monounsaturated fatty acids and polyphenols like hydroxytyrosol, which have complementary antioxidant and anti-inflammatory properties to resveratrol. The combination may provide enhanced protection against age-related diseases [104,106]. EVOO triggers an increase in glucuronide conjugates of resveratrol, while red wine increases sulfate metabolites. The combination may modulate resveratrol’s metabolic profile, potentially enhancing its bioactivity [104].

In conclusion, the development of nanoparticle-based delivery systems and the combination of resveratrol with EVOO represent promising strategies to overcome the limitations of resveratrol and enhance its therapeutic potential. Further research is needed to optimize these approaches and translate them into clinical applications.

## 5. Conclusions

This article explored the protective roles of resveratrol and EVOO against age-related diseases, highlighting their unique properties and synergistic benefits. Both resveratrol and EVOO exhibit significant neuroprotective properties through their antioxidant and anti-inflammatory activities, which help combat oxidative stress and inflammation—critical factors in neurodegenerative diseases like Alzheimer’s. Resveratrol and EVOO contribute to cardiovascular health by improving lipid profiles, enhancing endothelial function, and reducing inflammation, thereby lowering the risk of heart disease. However, more well-designed clinical trials are required to assess the long-term effects of resveratrol and extra virgin olive oil on health and cognitive functions in diverse populations.

Resveratrol has low bioavailability, which may limit its effectiveness. Although recent developments in delivery systems, such as polymeric nanoparticles, liposomes, and solid lipid nanoparticles, have shown promise in enhancing the solubility and stability of resveratrol, addressing its limitations, combining resveratrol with EVOO may improve the bioavailability of resveratrol due to the lipid matrix provided by EVOO, which aids in its absorption and metabolism.

The literature review indicates that both resveratrol and EVOO possess significant health benefits, particularly in the context of aging and age-related diseases. Their antioxidant, anti-inflammatory, and neuroprotective properties highlight their potential as protective agents. The combination of these two compounds may offer enhanced therapeutic effects, making them a promising strategy for promoting health and longevity. Furthermore, advances in delivery systems for resveratrol can potentially overcome its limitations, improving its clinical application.

Future research in this area should focus on conducting well-designed clinical trials to assess the long-term effects of resveratrol and EVOO, both individually and in combination, on cognitive function and overall health in diverse populations; investigating the specific molecular mechanisms through which resveratrol and EVOO exert their protective effects, particularly regarding their interactions with metabolic pathways and cellular signaling; exploring innovative formulation strategies that enhance the stability and bioavailability of resveratrol, including the use of novel delivery systems and combinations with other bioactive compounds; evaluating the impact of dietary interventions incorporating resveratrol and EVOO on health outcomes, including their effects on biomarkers of aging, inflammation, and oxidative stress. By addressing these areas, future research can further elucidate the potential of resveratrol and EVOO as effective interventions for promoting health and preventing age-related diseases.

## Figures and Tables

**Table 1:** Synergetic effects of resveratrol and EVOO on age-related disorders.

Aging Hallmark	Resveratrol Effect	EVOO Effect
*Molecular level*		
Genomic instability	PPAR α and SIRT6 (LINE-1 retrotransposon blockade)	LINE-1 hypomethylation inhibition
Telomere shortening	Pro-telomerase activity	Adherence to a diet with olive oil correlates with increased telomere length in certain populations
Epigenetic alteration	Reduces DNMT activity and their mRNA levels in breast cancer cells, leading to changes in DNA methylation; May modulate HDAC activity	Affection of miRNA expression; DNMT inhibition; histones deacetylases expression inhibition, histones acetylation inhibition
Loss of proteostasis	SIRT1 and SIRT5 activation	SIRT1 and mTOR activation
Compromised autophagy	p38 MAPK activation	MAPK activation
Mitochondrial dysfunction	PGC-1α activation, ROS inhibition	Alleviates oxidative stress
*Cellular level*		
Cellular senescence	Chronic inflammation reduction through NF-κB and NLRP3 inhibition	Inhibition of NF-κB, COX-2, LOX and enhancement of Nrf-2 signaling
Stem cell exhaustion		
Altered intercellular communication	Restore BBB integrity, reduces Aβ levels	Reduced expression of adhesion molecules, Aβ and tau phosphorylation, improved brain connectivity
*Systemic level*		
Nutritional dysregulation	Mimics caloric restriction, reduces blood glucose, improves insulin sensitivity	Improved lipid homeostasis
Age-related diseases	Improved cognitive performance, memory enhancement, reproductive health, immunometabolism amelioration, reduced cerebrovascular and cardiovascular risks, cancer and neurodegeneration prevention, lifespan extension	

Note: EVOO—extra virgin olive oil; PPARα—peroxisome proliferator-activated receptor alpha; SIRT—sirtuin; DNMT—DNA methyltransferase; mTOR—mammalian target of rapamycin; MAPK—mitogen-activated protein kinase; PGC-1α—peroxisome proliferator-activated receptor gamma coactivator 1-alpha; ROS—reactive oxygen species; NF-κB—nuclear factor κB; NLRP3—NLR family pyrin domain containing 3; COX-2—cyclooxygenase 2; LOX—lipoxygenase; Nrf-2—nuclear factor erythroid 2-related factor 2; BBB—blood–brain barrier; Aβ—amyloid-beta.
